# Exploring factors influencing farmers’ health self-assessment in China based on the LASSO method

**DOI:** 10.1186/s12889-024-17809-2

**Published:** 2024-01-31

**Authors:** Mingze Wu, Shulin Zeng

**Affiliations:** 1 College of Economics and Management, South China Agricultural University, Guangzhou, 510642 China; 2 Qidong Hospital of Traditional Chinese Medicine, Nantong, 226200 Jiangsu China

**Keywords:** Farmers’ health, Self-assessment of health, Dietary knowledge, LASSO method

## Abstract

As the main force and practice subject of rural revitalisation, farmers' health is intricately linked to agricultural production and the rural economy. This study utilizes open data from the 2015 China Nutrition and Health Survey and employs the Least Absolute Shrinkage and Selection Operator (LASSO) method to explore the factors influencing farmers' self-assessment of health. The findings reveal that education level, proactive nutrition knowledge seeking, healthy dietary preferences and habits, and the use of clean cooking fuel positively impact farmers' health self-assessment. Conversely, age, history of illness or injury, and participation in medical insurance negatively affect their self-assessment. Furthermore, factors influencing farmers' health self-assessment exhibit heterogeneity across regions. Our findings suggest that promoting health education, disseminating nutritional dietary knowledge, and enhancing rural household infrastructure play an important role in improving farmers' self-evaluation of health. Therefore, policymakers should design more targeted health interventions and infrastructure improvement plans based on farmers' self-assessment of health and the level of regional economic development.

## Introduction

Health is recognized as a fundamental component of human capital, playing a crucial role in both individual comprehensive development and national advancement, with profound implications for workforce participation and labour productivity [1–3]. Naturally, the health of individuals is closely intertwined with regional development levels. According to the latest statistics from the World Bank, as of the end of 2022, the global population has surpassed 8 billion people, with approximately 4.3 billion residing in rural areas, predominantly concentrated in impoverished regions (source: World Bank, 2023). Urbanisation rates reached 56.16% worldwide in 2020, with developed countries like the United States and Europe exceeding 80%, largely completing their urbanisation process [4]. China, as the largest developing and populous country in the world, has undergone significant urbanisation progress, marked by a reduction in permanent rural residents from 790 million in 1978 to 490 million in 2022 (source: National Bureau of Statistics, 2023). However, farmers still comprise a substantial proportion (34.8%) [5]. While health issues among urban residents and the elderly have garnered widespread scholarly attention [6–8], a notable disparity persists between rural and urban areas, encompassing infrastructure, healthcare, environmental pollution, food safety, and economic conditions. Consequently, the health of farmers, as crucial component of the primary industry's human capital, warrants increased consideration. It is of great practical significance and far-reaching social influence to study the factors influencing farmers' health.

Various disciplines emphasize different factors influencing individual health. In sociological studies, many scholars have verified the impact of factors such as education level, age, income, working environment and living conditions on people's health [9, 10]. However, economists attribute health heterogeneity to regional economic disparities, social development, institutional culture, and social welfare [11–13]. From the perspective of health medicine, factors affecting people's health include biological heredity, lifestyle, dietary preferences, interpersonal communication and emotions [14–17]. Some scholars even believe that direct behavioural and biomedical interventions can improve the health of residents in general [18]. In addition, physical education studies pay more attention to incorporating factors affecting physical health into the analysis framework from the perspective of ecological models of health behaviour [19]. A review of existing research shows that most of the literature focuses on the health status of urban residents, and health improvement among elderly and vulnerable groups.

Several crucial questions underlie our analysis. What is the health status of Chinese farmers amidst the backdrop of rapid urbanisation? Have their subjective health ratings improved compared to economically disadvantaged years? What factors significantly influence farmers' self-assessment of health? Are there important regional differences in those key influences? How can these influential factors be systematically identified through scientific means? Furthermore, what targeted recommendations should be proposed to policymakers to enhance the health status of farmers? To what extent can overall improvements in the health status of farmers, regarded as vital human capital, narrow the gap between urban and rural areas? However, we know very little about these concerns at this time, a more in-depth exploration is necessary to understand these issues.

Effective measurement and identification of individual health conditions are vital for policymakers to develop targeted interventions and implement policies to improve overall well-being. The literature has carried out much research on individual physiological health and mental health from both objective and subjective dimensions. Objective indicators, such as blood pressure, blood sugar, blood lipids, and BMI, provide insights into respondents' physical health [20]. Quality of life questionnaires such as the SF-36 and EQ-5D offer a comprehensive evaluation of the elderly people's and patients' physical health, mental health, emotion, and social participation [21, 22]. The Depressive Symptom Assessment Scale and the Anxiety Symptom Assessment Scale are widely used to measure an individual's mental health [23, 24]. Moreover, health self-assessment, a subjective measure of individuals' health perception, plays a crucial role in understanding residents' health and well-being. In this method, the simple Likert scale scoring method allows individuals to independently rate their health based on subjective feelings, and thanks to its simplicity, it has been widely adopted by scholars [25, 26]. Leveraging available data, this study employs respondent-reported health self-assessment as the dependent variable.

This study aims to accurately identify crucial factors related to farmers' health through the application of LASSO regression, a supervised machine learning method. By analysing multiple variables encompassing dimensions of farmers' individual characteristics, health literacy, dietary preferences, and cooking fuel choice, we aim to attain a comprehensive understanding of the diverse factors influencing health among individuals. The findings of this study provide valuable guidance and decision-making support for policymakers and health professionals. This includes tailored health interventions for different groups, optimized allocation of medical resources, implementation of preventive and early care measures, and enhancement of health services. This paper contributes to the literature by introducing machine learning algorithms, in contrast to traditional methods like linear regression and Poisson regression [26]. This enriches the research methodology, reduces redundant variables, and enhances the model's explanatory and generalisation capabilities.

The remainder of this paper is organized as follows. "Research methodology" section presents the research methods.  "Data description" section introduces the data in this paper, including index construction, sample processing, and descriptive statistics of variables. The empirical results are presented and discussed in "Results and discussion" section. The last section comprises the conclusion and implications.

## Research methodology

Logistic models are widely used for binary discrete dependent variable problems. When all the variables in the study are included in the regression model to be fitted, it is prone to the risk of multicollinearity and overfitting. To screen the factors affecting farmers' health self-assessment more scientifically and objectively, the present study was conducted with the help of LASSO method for variable screening. The LASSO algorithm implements feature selection and model parameter contraction by adding an L1 regularisation term to the loss function. This method, proposed by Tibshirani in 1996, is essentially traditional least squares estimation with penalty factors. Using the absolute value function of the model coefficients as a penalty effectively reduces the weights of the unimportant features and compresses their coefficients to zero, which leads to variable selection and parameter estimation, and results in a more refined model [27]. In contrast to conventional variable selection approaches like stepwise regression, ridge regression, principal component regression, partial least squares regression, and others, the LASSO method stands out for its robust utility in data analysis and feature selection. This method boasts distinct advantages, including heightened predictive accuracy, enhanced model interpretability, and computational simplicity [28].

LASSO is a method of logistic modelling using the LASSO method of selecting independent variables to rule out the omission of variables due to preconceived notions, as well as by scaling down the bias estimates, which in turn removes linear relationships between variables [29]. Assuming that there are independently and identically distributed observations $({X}^{i}, {y}_{i})$, $i=\mathrm{1,2},\dots ,n$, $j=\mathrm{1,2},\dots ,p$, the conditional probability expression for the Logistic model is as follows:

1 $$ln\frac{P({y}_{i}=1|{X}^{i})}{1-P({y}_{i}=1|{X}^{i})}=\eta \beta \left({X}^{i}\right)={\beta }_{0}+\sum_{j=1}^{p}{x}_{ij}{\beta }_{j}$$

where ${X}^{i}$ and ${y}_{i}$ are the independent and dependent variables of the model, respectively. $P$ denotes the probability of the model and $\beta$ is the coefficient.

The coefficient estimates $\widehat{\beta }$ in the LASSO model can be written as Eq. (2):

2 $$\widehat{\beta }=argmin\sum_{i=1}^{n}\left\{{y}_{i}\eta \beta \left({X}^{i}\right)-ln\left\{1+exp\left[\eta \beta \left({X}^{i}\right)\right]\right\}\right\}+\lambda \sum_{j=1}^{p}\left|{\beta }_{j}\right|$$

where $\lambda$ is a non-negative tuning parameter that determines the degree of compression of the LASSO model coefficients. As $\lambda$ increases, the coefficient estimates of each independent variable are gradually compressed, and some independent variable coefficients will be compressed to zero, resulting in a streamlined model with fewer independent variables.

In this study, LASSO analyses were carried out using the "*glmnet*" package of R software. The determination of the optimal tuning parameter $\lambda$ directly affects the number of variables, the choice of type, and the results of the parameter estimation. There are several main approaches to the selection of $\lambda$ in existing studies: Bayesian Optimisation, Cross-Validation, Grid Search, and Random Search [30–33]. Cross-validation was used to determine the $\lambda$ in this study. The specific steps are as follows: first, make the fold assignment by randomly splitting the data into K approximately equal groups, which will be used to estimate the prediction error for each value of $\lambda$. Second, withhold one of the K folds and fit each candidate model to the remaining K − 1 folds, denoted ${f}^{k}$. Third, compute the prediction error of each candidate model fitted in the previous step over the withheld fold, and repeat multiple times until each fold of data has been withheld. Finally, aggregate the prediction errors obtained over the K folds, and the optimal value of $\lambda$, is chosen to be the value that corresponds to the candidate model with the smallest aggregated prediction error [34]. Since k is often taken to be 10 in practice, it can be called ten-fold cross-validation [33]. The prediction error for ten-fold cross-validation can be expressed as:

3 $$CV\left(f\right)=\frac{1}{N}\sum_{i=1}^{N}L[{y}_{i},{f}^{k\left(i\right)}({x}_{i})]$$

where $k\left(i\right)$ denotes the indicator function of the $N$ samples in which observation $i$ belongs to the $k$ th $(k=\mathrm{1,2},\dots ,K)$ data; and ${f}^{k}$ denotes the model fitted using the exclusion of the $k$ th data. Assuming that fitting a set of models containing tuning parameters is ${f}^{k}(x,\lambda )$, it is defined as:

4 $$CV\left(f,\lambda \right)=\frac{1}{N}\sum_{i=1}^{N}L[{y}_{i},{f}^{k\left(i\right)}({x}_{i},\lambda )]$$

where $CV\left(f,\lambda \right)$ denotes a test error curve that varies with $\lambda$. The $\lambda$ that minimises $CV\left(f,\lambda \right)$ is the tuning parameter of the LASSO model.

## Data description

### Indicator construction

Building upon previous literature, this study aims to explore the potential factors influencing individuals' subjective health self-assessment. To obtain quantifiable micro-level data and refer to the indicator construction of existing studies [35], the present research decomposes the perspectives of "Individual level" "Family level" "Health behaviour" and "Dietary preferences" into seven categories of influencing factors, such as "Individual conditions" and "Positive health behaviours". These factors are further refined into 24 specific indicator variables. These 24 variables constitute the indicator system of this study on the influencing factors of subjective health self-assessment among a nationally representative sample of farmers (Table 1).

**Table 1:** Indicator system of factors influencing farmers' health self-assessment

Research perspectives	Influence factors	Variable
Individual level	Individual conditions	Gender
		Age
		Education
		Marry
		Sick
		Folk doctor
Health behaviour	Positive	Dietary guideline
		Nutrition knowledge
		Insurance
	Negative	Cigarettes
		Alcohol
Dietary preferences	Unhealthy	Fast food
		Salty foods
		Soft drinks
	Healthy	Fruits
		Vegetables
Family level	Hardware	Internet
		Filter water
		Flush toilet
	Cooking fuels	Coal
		Electricity
		Liquefied petroleum gas (LPG)
		Natural gas
		Wood

### Data sources and sample processing

The data used in this work are from the China Nutrition and Health Survey (CHNS). The survey was organized by The Chinese Center for Disease Control and Prevention, The National Institute of Nutrition and Food Safety, and The Carolina Population Center at the University of North Carolina at Chapel Hill. The CHNS survey was initiated in 1989 and has been conducted in 10 waves. The survey adopts the multi-stage stratified cluster random sampling method with the following steps: First, the simple random sampling method was used to select a total of nine provinces (Liaoning, Heilongjiang, Jiangsu, Shandong, Henan, Hubei, Hunan, Guangxi, and Guizhou) and three municipalities directly under the Central Government (Beijing, Shanghai, and Chongqing) scattered in eastern, central, and western regions of China. Second, different counties in each province were stratified according to differences in residents' income levels, and after weighting, four counties (including one high-income, two middle-income, and one low-income) were randomly selected from each of the above provinces as representatives of the rural sample; for the urban areas. The capital of each province and a low-income city were selected as urban samples. Next, three administrative villages were randomly selected from each county. Finally, 220 communities were selected from the rural and urban samples, with approximately 20 family households interviewed in each community, for a total of 4,400 households included in the survey. The CHNS database has been widely used in research in related fields such as medicine, economics and management due to its strong rigour and representativeness [36–38]. In our study, cross-sectional data from 2015 was selected, which is the latest year of data currently available for open access and could more accurately reflect the current physical health status and dietary preferences of Chinese individuals.

The aim of this study is to explore the significant factors influencing health self-assessment among farmers. First, we focus on smallholder farmers who permanently reside outside of city areas and are engaged in agricultural production activities. Second, considering that the variables related to dietary knowledge and dietary preferences in the questionnaire are applicable to residents aged 12 and above, we further excluded individuals below the age of 12 who were minors. Finally, after excluding the samples with missing data for key variables, we obtained a total of 10,115 valid samples for 2015.

Considering that the "*glmnet*" package in R software is primarily used for regression problems involving continuous dependent variables and binary categorical variables, the "In the last twelve months, how would you define your state of health" question in the questionnaire, which implemented a 5-point Likert scale, was transformed into a binary variable in our study: scores of 1 to 3 ("very poor" "poor" and "fair") were classified as "0 = unhealthy", while scores of 4 to 5 ("good" and "very good") were classified as "1 = healthy".

### Descriptive statistics

According to Table 2, among the surveyed respondents, over half of the farmers reported having good physical health. The majority of respondents were female, with an average age exceeding 50 years, and had a low level of education, with only primary school qualifications. Most of the farmers in the sample were married, accounting for approximately 83.6%. Approximately 12.6% of individuals reported experiencing illness or injury in the past month, with a relatively low proportion seeking medical care from informal practitioners, accounting for only about 5%. We found that only about 20% of farmers demonstrated proactivity in terms of their knowledge and acquisition of nutritional dietary knowledge. Almost all farmers had purchased health insurance. The proportions of individuals who smoke and consume alcohol are 27.1% and 27.4%, respectively. In terms of dietary intake, we found that the majority of farmers exhibited good dietary preferences, preferring fruits and vegetables over high-fat, high-salt "junk food." The proportion of farmers using the internet was relatively low, less than 30%. Approximately 12.4% of rural households had access to filtered drinking water, while over 60% of households had flush toilets. The highest proportion of households used liquefied gas for cooking, followed by electricity, and then cleaner natural gas. However, a small portion of households still rely on wood and coal for cooking.

**Table 2:** Variable definitions and descriptive statistics

Variable	Definition	Mean	Std. Dev
Health self-assessment	1 = good health, 0 = bad health	0.506	0.500
Gender	1 = male, 0 = female	0.474	0.499
Age	Respondents’ age	50.414	16.555
Education	Respondents’ education level	1.912	1.441
Marry	Currently married? 1 = yes, 0 = otherwise	0.836	0.370
Sick	1 = if respondents have been sick or injured in last 4 weeks, 0 = otherwise	0.126	0.332
Folk doctor	1 = if respondents visit a folk doctor last year, 0 = otherwise	0.054	0.227
Dietary guideline	1 = know about the Dietary Guidelines for Chinese Residents, 0 = otherwise	0.199	0.399
Nutrition knowledge	1 = proactively look for nutrition knowledge, 0 = otherwise	0.208	0.406
Insurance	1 = if respondents have medical insurance, 0 = otherwise	0.975	0.156
Cigarettes	Have you ever smoked cigarettes? 1 = yes, 0 = otherwise	0.271	0.445
Alcohol	Drank beer/alcohol last year? 1 = yes, 0 = otherwise	0.274	0.446
Fast food	Likes fast food? (KFC, pizza, hamburgers, etc.) 1 = dislike very much, 5 = like very much	2.037	0.786
Salty foods	Likes salty snack foods? (potato chips, pretzels, etc.) 1 = dislike very much, 5 = like very much	2.163	0.809
Soft drinks	Likes soft/sugared drinks? 1 = dislike very much, 5 = like very much	2.567	0.930
Fruits	Likes fruits? 1 = dislike very much, 5 = like very much	3.622	0.744
Vegetables	Likes vegetables? 1 = dislike very much, 5 = like very much	3.788	0.657
Internet	1 = if respondents use the internet, 0 = otherwise	0.282	0.450
Filter water	1 = if respondents filter water before drinking, 0 = otherwise	0.124	0.329
Flush toilet	1 = have a flush toilet at home, 0 = otherwise	0.640	0.480
Coal	1 = use coal fuel for cooking, 0 = otherwise	0.066	0.249
Electricity	1 = use electricity for cooking, 0 = otherwise	0.303	0.459
LPG	1 = use liquefied petroleum gas for cooking, 0 = otherwise	0.346	0.476
Natural gas	1 = use natural gas for cooking, 0 = otherwise	0.170	0.375
Wood	1 = use wood, sticks/straw, etc. for cooking, 0 = otherwise	0.105	0.306

## Results and discussion

**Fig. 1:**
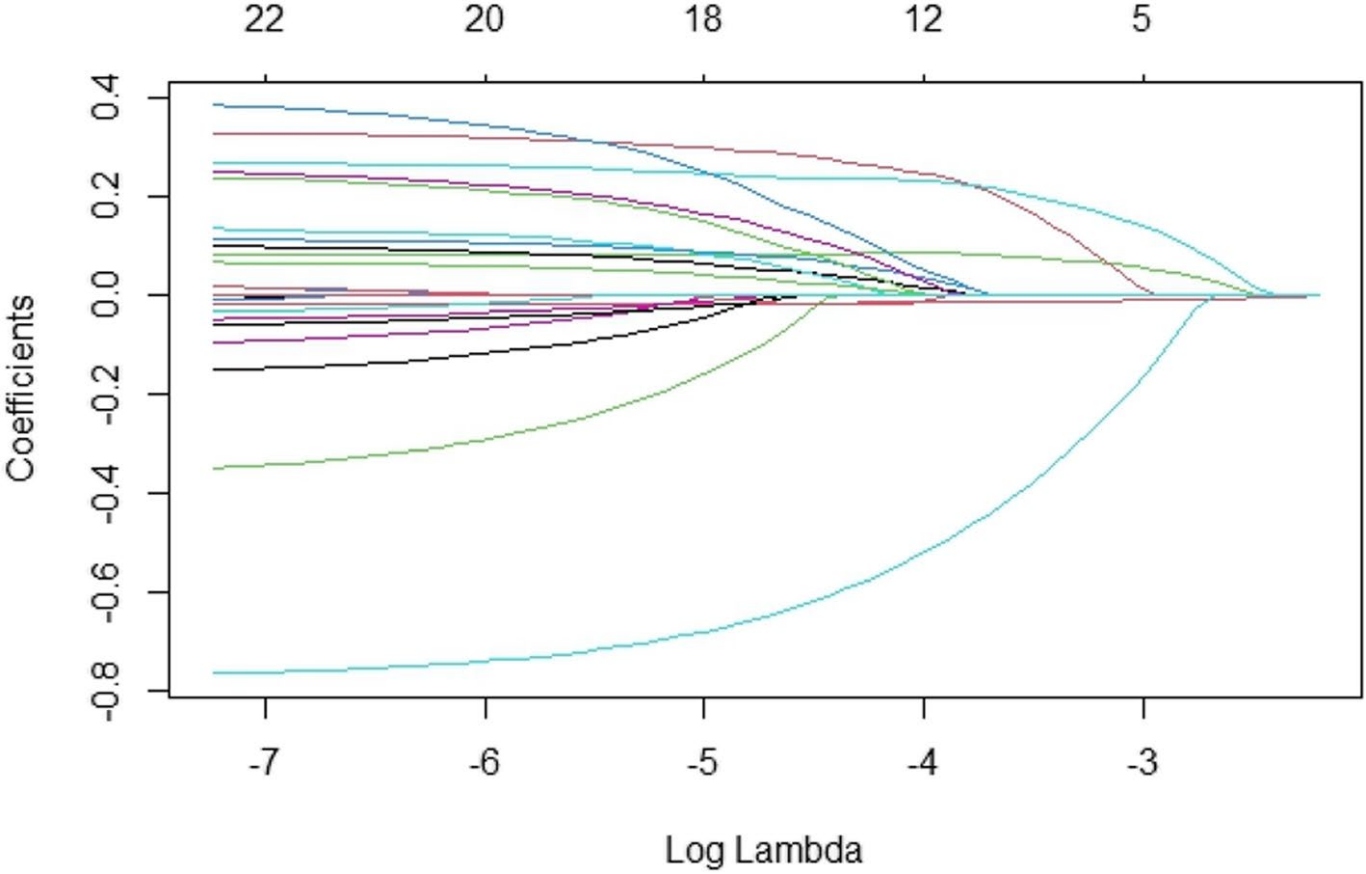
Variable selection path for the LASSO model

To comprehensively analyse the factors influencing subjective health self-assessment among representative farmers in China, this study selected 24 micro-level influencing factors, including gender, age, education, marital status, and others. Logistic regression was initially employed, and subsequently, the LASSO method was applied to select variables and estimate parameters from the 24 chosen factors. The dynamic process of variable selection using the LASSO method is depicted in Fig. 1.

**Fig. 2:**
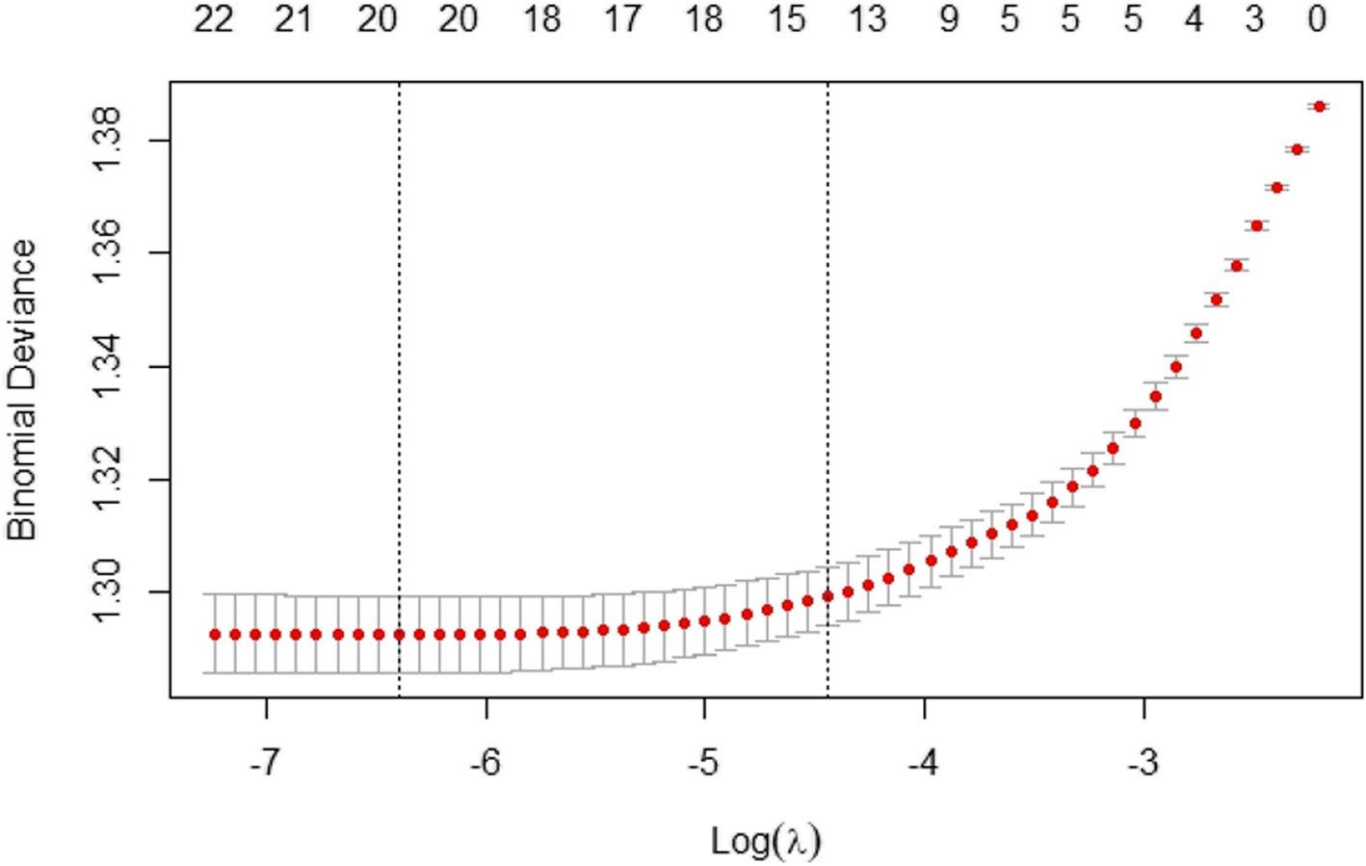
Correspondence between λ and the number of independent variables

Figure 2 represents the graph generated by the model through cross-validation. The vertical axis represents the magnitude of model bias, while the horizontal axis represents the values of the tuning parameter $\lambda$. The two vertical lines in Fig. 2 from left to right represent the $\lambda$ with the minimum average error (lambda.min) and the maximum $\lambda$ with the average error within one standard deviation (lambda.1se). When lambda.min is selected, the model can be allowed to fit more features. This choice is usually suitable for paying more attention to the predictive performance of the model, but not too much about the complexity of the model; the disadvantage is that there may be a risk of overfitting. When lambda.1se is selected, the model more strongly penalizes unimportant variables, leading to the selection of fewer features and limiting the number of non-zero coefficients in the model, thereby reducing the risk of overfitting [27, 32]. Based on the results of cross-validation and the sample size, this study prioritizes the interpretability of the model. Therefore, lambda.1se is selected as the optimal $\lambda$. With this $\lambda$ value, the model ultimately removes 10 variables and retains 14 variables.

The second column of Table 3 shows the regression results of the logistic model. The results showed that age, illness or injury in the last month, having medical insurance, liking salty food, and flush toilet at home had statistically significant negative effects on farmers’ self-assessment of health; while education level, knowing about dietary guidelines, proactively looking for nutrition knowledge, drinking alcohol, liking fruits and vegetables, internet usage, filtering water before drinking, cooking with LPG and natural gas had statistically significant positive effects on farmers’ self-assessment of health. However, there are some differences between the variables selected by the LASSO method and the statistically significant regression results in the Logistic model. The variables "salty foods" and "flush toilet" showed statistical significance in the Logistic regression analysis. However, these variables were not selected by the LASSO method, suggesting that they no longer exhibit a statistically significant relationship with farmers’ health self-assessment. Interestingly, the coefficient of the variable "use electricity for cooking" has changed from positive to negative. Next, we will proceed with the analysis using the variables that were selected through the LASSO method.

**Table 3:** The Logistic and LASSO estimation results

Variable	Logistic	LASSO
Gender	-0.029(0.056)	
Age	-0.020***(0.002)	-0.206
Education	0.085***(0.018)	0.084
Marry	-0.024(0.061)	
Sick	-0.779***(0.068)	-0.609
Folk doctor	-0.107(0.095)	
Dietary guideline	0.104*(0.063)	0.041
Nutrition knowledge	0.334***(0.062)	0.277
Insurance	-0.375***(0.138)	-0.005
Cigarettes	0.035(0.061)	
Alcohol	0.147**(0.059)	0.038
Fast food	-0.053(0.041)	
Salty foods	-0.065*(0.039)	
Soft drinks	0.024(0.026)	
Fruits	0.069**(0.035)	0.021
Vegetables	0.117***(0.040)	0.067
Internet	0.271***(0.060)	0.237
Filter water	0.261***(0.065)	0.104
Flush toilet	-0.164***(0.048)	
Coal	0.110(0.217)	
Electricity	0.099(0.204)	-0.016
LPG	0.349*(0.203)	0.072
Natural gas	0.501**(0.207)	0.148
Wood	0.060(0.212)	
Constant	0.461(0.306)	-
Chi-square	858.866***	
Observations	10,115	

Standard errors in parentheses. Significance level: *** *p* < 0.01, ** *p* < 0.05, * *p* < 0.1

We conduct a specific analysis from the following four aspects. At the individual level, as age increases, various physiological functions of the body gradually decline. This natural aging process often leads individuals to perceive their health as less robust compared to younger people [8]. However, a study has shown that compared to individuals aged 65 to 90, elderly people over 90 tend to reverse their perception of health and exhibit a better health self-assessment. This phenomenon can be attributed to the heterogeneity of the aging process and the development of adaptive mechanisms [39]. A higher level of education has a positive impact on farmers' subjective health self-assessment. This can be attributed to several factors. Farmers with higher education levels are more likely to have opportunities to access health knowledge and information, understand how to maintain health and prevent diseases, and have the ability to utilize healthcare resources effectively. Additionally, individuals with higher education levels may possess positive psychological factors such as self-confidence and an optimistic attitude towards life. These psychological factors help individuals cope with challenges and stress in life, resulting in a positive impact on their health status [40, 41]. Therefore, individuals with higher education levels are more likely to provide higher health self-assessments. Conversely, [26] found, through their research on women in developing countries like Brazil, that lower educational attainment is a predictive factor for lower self-assessments of health. It is not surprising to find that farmers who have reported experiencing illness or injury in the past month would have a lower self-assessment of their health. However, it is important to note that this does not necessarily imply that they would consistently provide an unhealthy self-assessment when asked about their health status in the future. Individuals who have experienced recent illness or injury may temporarily perceive their health as poor, but as they recover and their health improves, their self-assessment may also change.

In terms of health behaviours, farmers who possess knowledge about dietary guidelines and actively seek nutrition information tend to have higher self-assessments of their health. On the one hand, farmers' active pursuit of nutrition and health knowledge can motivate them to adopt healthier dietary behaviours. This may include increasing the consumption of fruits, vegetables, and whole grains while reducing the intake of high-sugar, high-salt, and high-fat foods [42]. Additionally, dietary knowledge empowers farmers to understand the nutritional content of different foods, promoting dietary diversity. This is crucial for preventing malnutrition, reducing the risk of chronic diseases, and improving overall health [43]. On the other hand, acquiring dietary knowledge enhances farmers' awareness of food safety and hygiene practices [44]. They can learn how to select and store food properly, avoid food poisoning, and employ appropriate cooking and processing techniques to preserve the nutritional elements of food. We found unexpected results regarding the impact of farmers' participation in medical insurance and alcohol consumption on their health self-assessments. Farmers' participation in medical insurance was negatively associated with their subjective health assessments, while drinking alcohol had a positive impact on farmers' subjective health assessments, this finding contrasts with previous research by [43]. These surprising results emphasize the complexity of the relationship between these variables and health self-assessments among farmers. We agree that the possible reason for the unexpected results may be due to the endogeneity problem caused by reverse causality. For example, individuals with poorer health may be more likely to seek medical insurance. Likewise, the group categorized as healthy demonstrated a higher likelihood of alcohol consumption compared to the group classified as frail. Alcohol consumption may be closely associated with complex social networks, and participation in social activities can lead to positive emotional and mental health, which is reflected in health self-assessment [45]. Farmers with poorer health conditions tend to practice moderation in alcohol consumption and participate less in social activities. Conversely, farmers with better health conditions tend to consume alcohol more frequently and possess greater energy to engage in social activities.

Regarding dietary preference, farmers who preferred fruits and vegetables reported better health self-assessments. There may be a substitution effect between healthy dietary preferences and unhealthy dietary preferences. Fruits and vegetables are important food sources of high-quality vitamins and dietary fibre. Supplementation of these nutrients helps the body function properly, promotes intestinal health, maintains digestive system function, and strengthens immunity, thereby improving the overall health of the individual [46].

At the family level, the subjective health self-assessments of farmers were positively influenced by Internet use. The widespread popularity of the Internet has significantly reduced the information gap between rural and urban areas, allowing farmers to access health-related knowledge more easily, and providing a platform for them to communicate and share experiences with others. At the same time, the Internet enables farmers to obtain health services remotely, such as online consultation and purchase of medicines [47, 48]. The convenient and efficient health service methods offered by Internet platforms enable farmers to effectively manage health problems and enhance their perception and evaluation of their health. Our study is consistent with [49], suggesting that unsanitary water facilities can lead to pathogen transfer and cause diseases, and the direct consumption of unclean water may have a negative impact on farmers' health. Apart from removing suspended solids from the water source, pre-drinking water filtration also has the potential to reduce the presence of harmful substances such as bacteria, viruses, and pesticide residues, which significantly affects human health [50]. Especially in most developing countries, the practice of filtering water before drinking should draw sufficient attention from relevant authorities and organisations. The use of electricity reduces farmers' self-assessment of health; while the use of clean fuels like LPG and natural gas as the primary cooking fuel has a positive effect on farmers' self-assessment of health [51]. Traditional fuels such as coal and firewood generate significant amounts of smoke and harmful gases, including PM2.5, PM10, and carbon monoxide, during the cooking process [52, 53]. These pollutants not only pose severe threats to the ecological environment, but also cause long-term irreversible damage to the respiratory and cardiovascular systems when inhaled by individuals [54]. In contrast, the use of clean fuels can reduce the health damage caused by the combustion of traditional fossil fuels, provide a safe cooking environment, and feature convenient operations. Additionally, it can also reduce greenhouse gas emissions, which has a positive impact on mitigating global warming and promoting environmental sustainability [50].

Considering the economic differences among the sample regions, we further divided the samples according to the South and the North to study the subjective evaluation of farmers' health status in different areas. The results are shown in Table 4 (The appendix shows the graphical results of cross-validation). Through comparison, we find that there are significant differences in the factors affecting the health self-assessment of farmers in the North and the South. Southern farmers who visited a folk doctor last year had lower health perceptions than their northern counterparts. Knowledge of dietary guidelines may only have a significant positive impact on the health of farmers in the South. Preferences for fruits and vegetables seem more likely to enhance the subjective health ratings of southern farmers. Filtering water sources only improved perceptions of health among southern farmers. The use of coal fuel reduced the perceived health of farmers in the North, while the burning of wood significantly diminished the perceived health of farmers in the South. The use of LPG was only able to improve the health perception of farmers in the North. The regional groupings help us identify the key factors influencing farmers' health self-assessment from a more comprehensive perspective.

**Table 4:** Regional heterogeneity of the LASSO method

Variable	Total	South	North
Gender			
Age	-0.206	-0.016	-0.017
Edu	0.084	0.059	0.089
Marry			
Sick	-0.609	-0.610	-0.475
Folk doctor		-0.062	
Dietary guideline	0.041	0.064	
Nutrition knowledge	0.277	0.139	0.363
Insurance	-0.005		
Cigarettes			
Alcohol	0.038	0.009	0.031
Fast food			
Salty foods			
Soft drinks			
Fruits	0.021	0.031	
Vegetables	0.067	0.178	
Internet	0.237	0.169	0.383
Filter water	0.104	0.183	
Flush toilet			
Coal			-0.201
Electricity	-0.016	-0.134	
Liquified petroleum gas	0.072		0.019
Natural gas	0.148	0.065	0.079
Wood		-0.038	
Observations	10,115	6175	3940

## Conclusions and implications

### Conclusions and limitation

This study is based on micro-survey data from 10,115 representative Chinese farmers, selects 24 variables across four dimensions and employs the LASSO method to empirically study the influencing factors of farmers' health self-assessment. The results indicate the following: (1) education level has a positive impact on farmers' health perception, while age and recent experience of illness within the past month have a negative impact. (2) Actively seeking dietary and nutritional knowledge, and having a preference for healthy eating habits such as consuming fruits and vegetables, positively influence farmers' health self-assessment. Surprisingly, alcohol intake shows a positive correlation with health self-assessment, while a majority of farmers with medical insurance report an unhealthy physical condition. (3) Internet usage and filtering water sources before drinking show a positive correlation with farmers' health self-assessment. In terms of cooking fuel used in households, the adoption of clean LPG and natural gas demonstrates a positive significance in farmers' health. (4) Significant differences exist in the factors influencing the health perceptions of farmers in the North and South. Southern farmers appear to be more proactive in understanding dietary guidelines and consuming fruits and vegetables, whereas farmers in the North seem to be more inclined to adopt LPG, resulting in more positive health feedback.

This paper employs machine learning algorithms to advance research in the field of health, enabling a more precise identification of factors influencing farmers’ subjective health self-assessment. This approach makes a meaningful contribution to the existing literature and provides technical methodological support for future researchers exploring the identification of influencing factors. Certainly, it is necessary to acknowledge the limitations of this study, in particular the inclusion of only 24 factors in the model for variable screening. The full advantages of the algorithm might be realized only when a broader set of variables is considered. In future empirical analyses, the collection of more comprehensive data that incorporates a broader range of factors influencing farmers' health into the model for selection could further optimize the existing research conclusions. In addition, this study did not consider causal inferences about the mechanisms affecting the health of smallholder farmers, which could be a direction for further expansion in the future.

### Implications

The results of this study provide several important implications. First, the government should strengthen health promotion and psychological counselling in rural areas, offering easily understandable and actionable health knowledge, encompassing nutrition, disease prevention, personal hygiene, and other informative aspects. Health knowledge can be disseminated and popularized through channels such as village committees and health stations, utilizing methods such as pamphlets and training lectures. Additionally, efforts should be made to provide psychological support, especially for vulnerable groups, to enhance their social support and sense of participation. This can contribute to improving their subjective health evaluation. Second, policymakers bear the responsibility of actively enhancing health awareness and promoting healthy behaviours among the farmers. Through community health promotion activities, media campaigns, and other communication channels, farmers can be motivated to adopt positive and healthy dietary habits, fostering changes in unhealthy lifestyles. For example, encouraging the diversification of diets, increasing dietary fibre intake, and limiting the consumption of high-salt and high-fat foods. These measures aim to improve the health level and overall quality of life for farmers. Third, relevant stakeholders should strengthen infrastructure according to the needs of different regions. For example, the effective operation of electricity facilities should be enhanced in the South, while water filtration equipment and flush toilets should be strengthened in the North, which is essential to improving the living environment and sanitation of farmers, thereby helping to prevent the spread of diseases. Fourth, the government and relevant organisations should actively promote the use of clean fuels, such as LPG and natural gas, especially in impoverished rural areas. Measures such as providing subsidies, tax exemptions, or offering preferential policies can be implemented to encourage farmers to use clean fuels.

## Data Availability

The data supporting the findings of this study are available from CHNS (https://www.cpc.unc.edu/projects/china/data/datasets).

## Appendix

Results of cross-validation between the South and the North.
